# Voyages in Vacation.—XI

**Published:** 1888-12-08

**Authors:** 


### VOYAGES IN VACATION.—XI.

BY ONE IN SEARCH OF RESTFUL RESTORATIVES.

*(Continued from p. 135.)*

### RUSSIAN PALACES.

It will be gathered from the remarks made in a previous article (page 103, vol. V.) that our impressions of Russia were rudely upset on entering St. Petersburg. Indeed, the more we became acquainted with the condition of the business and the character and number of the palaces, both imperial and private, the more we were impressed with the feeling that there ought to be no doubt as to the stability of Russia.

A careful inspection would go far to convince the most sceptical that the loot in the Palaces alone ought to be of sufficient value to redeem the National Debt. It is by no means easy to obtain admission to the Winter Palace at the present time. Formerly passes were issued freely, but circumstances alter cases, and so it has been thought prudent to restrict the number as much as possible to the privileged few. No foreigner can obtain admission except by the intervention of the Ambassador of the country to which he belongs, and even his influential backing is not always sufficient to secure admission. Externally, the Winter Palace presents anything but an imposing or attractive appearance. It is a vast block of stone buildings, as may be gathered from the statement that the frontage is upwards of 450 feet, and the breadth represents a depth of quite 350 feet. It is four storeys in height, that is about eighty feet, and the whole of the stonework has been washed over like most of the buildings in St. Petersburg, with a warm-coloured dressing of some kind, which we were informed had to be renewed each spring. The principal entrance is from the Neva, by a fine flight of marble steps which lead to the State apartments.

Murray states, and we can well believe, that no court in Europe presents such a brilliant appearance as that of Russia when seen in the Winter Palace. Let any reader try to imagine the whole of this magnificent marble staircase brilliantly lighted, the steps covered with the richest of carpets, and on either side of each step two of the Imperial household in their splendid uniforms, with the guests ascending in an almost endless stream through the human avenue thus formed to meet the Czar and his brilliant court. The Nicholas and St. George's Hall, the Concert Room, the White Hall, and the Golden Hall are among the finest-proportioned apartments which it is possible to conceive. The White Hall must prove specially interesting to visitors, because they will there find the large collection of gold and silver-gilt dishes on which bread and salt have been presented to the Czar. These are the salt dishes referred to last week. The things which specially interested us were: (1) the pictures; (2) the drawing-room of the Empress Alexandra, with her dwelling-rooms; and (3) the study of Alexander II., and the rooms of Nicholas I.

The pictures consist mainly of battle scenes, represent all the great wars in which Russia has been engaged, and include many naval engagements from 1700 to the present time. There is a graphic power about many of these pictures which attracts and fixes the attention of the thoughtful, and we venture to think could they be placed at the disposal of the Peace Society and exhibited in order in every capital in Europe, the society would have secured an ally more powerful than any that can well be imagined. Of course to the military man and to the antiquary the weapons, ordnance, accoutrements, and other details are of supreme interest, and in many instances, owing to the great size of the pictures, a fair idea of the tactics pursued at different periods may be gleaned. The pictures embrace scenes which may be truthfully described as exhilarating or heart-breaking, as harrowing or encouraging, as horrible, cruel, or appalling, according to the circumstances of the particular engagements or the class of incident which the artist has had to depict. Taken altogether, the collection may be considered one of the most instructive of exhibitions, and one which every visitor should not fail to take occasion to inspect. In the Portrait Gallery and Field Marshal's Hall a large number of distinguished military and naval officers of various nations as well as those of Russia are placed, whilst the historian will no doubt spend some hours in the Romanoff Gallery, which contains the portraits of all the sovereigns of the reigning house since Michael Feodorovitch. Portraits of the consorts of each Emperor will here be found, and of course that of Peter the Great finds not one, but many places in this gallery.

Amidst all the grandeur and magnificence of the Winter Palace one was pleased to find the simple private apartment of the Empress Alexandra, consort of Nicholas I. These dwelling-rooms of hers are simplicity itself, and so is the study of Alexander II., where one is able in imagination to see the Emperor at home. His camp bed on which he died is carefully preserved here; and in a room below one is shown the hard camp bedstead, military cloak, sword, helmet, and slippers of Nicholas I., who died in that apartment. In each case these private rooms show the culture, the homeliness, and the domestic happiness of the inner life of these departed monarchs. We spent quite half-an-hour in the study of Alexander II., which contains proof after proof of his practical sympathy with the sufferings of his people, of his genuine interest in art and literature, as well as in military matters, and bears eloquent testimony to the keen interest which he took in the welfare, progress, and doings of his many relatives and friends. Not the least touching of these evidences is the small marble bust of his tutor, seated in a chair, which the Emperor always kept on his writing-table beside him, that he might be reminded of one who had such a strong influence on his character and life. The books of devotion, too, which these monarchs used bear evidence that they were their constant solace and help throughout their lives. We venture to think that these rooms and their contents will be regarded by most English people as the most interesting of the many enlightening sights they will meet with in St. Petersburg.

Yet these sovereigns could display most regal state on occasion. This fact is forcibly brought home to the visitor when he enters the drawing-room of the Empress Alexandra, which is still preserved as it was at the time when the consort of Nicholas I. was wont to hold the most brilliant receptions in these elegant apartments. The ceilings and doors are richly gilt, and attention is at once attracted to the walls by the interesting frescoes after Raphael, which form their chief ornament. The ceilings of these rooms, like those throughout the palace, are wonderful works of art, and must give many new ideas to those who make a study of artistic decoration. The furniture too, is chased to a degree, and no one can fail to notice the unique mantelpiece of malachite, with the elegant vases of the same expensive stone, as well as the brilliant candelabra of lapis lazuli, which are not the least attractive feature of this very fine drawing-room. We were also shown the rooms formerly occupied by H.R.H. the Duchess of Edinburgh, chiefly remarkable for the lowness of the ceilings and the plainness of the chintz-covered furniture. Another arrangement which struck our fancy was the raised platform which was railed off in a corner of some of the dwelling-rooms where the Royal party with their immediate attendants were in the habit of sitting. These spaces were all surrounded with flower-vases, which are filled with plants and flowers when the palace is occupied, and so help to form a most attractive and novel boudoir for the fortunate occupants. It is worthy of notice too, that every room is kept in the most perfect order, so that they can be made ready for occupation at a few hours' notice. This was the case in all the palaces, and the evidence it afforded of perfect supervision and excellent housekeeping said much for the efficiency of the staff employed.

It would, of course, be impossible to give an account of all the palaces in St. Petersburg, which are so numerous that we refrain from stating the precise number as being almost incredible. The present Emperor occupies the Anitchkoff Palace, which is situated in the Nevski Prospect. This palace is not very striking in appearance, and the only outward sign of its character is to be gathered from the presence of two sentry-boxes on either side of the entrance, which abuts on the pavement in the same way as the gates of Marlborough House open into Pall Mall.

*(To be continued.)*

